# Diaquabis­[3-(hydroxy­imino­)butanoato]nickel(II): a triclinic polymorph

**DOI:** 10.1107/S1600536810006306

**Published:** 2010-02-20

**Authors:** Valentina A. Kalibabchuk, Nikolay M. Dudarenko, Turganbay S. Iskenderov, Maria L. Malysheva, Elżbieta Gumienna-Kontecka

**Affiliations:** a Department of General Chemistry, O.O. Bohomolets National Medical University, Shevchenko Blvd 13, 01601 Kiev, Ukraine; b Department of Chemistry, Kiev National Taras Shevchenko University, Volodymyrska Street 64, 01601 Kiev, Ukraine; c Department of Chemistry, Karakalpakian University, Universitet Keshesi 1, 742012 Nukus, Uzbekistan; d Faculty of Chemistry, University of Wrocław, 14 F. Joliot-Curie Street, 50-383 Wrocław, Poland

## Abstract

The title centrosymmetric mononuclear complex, [Ni(C_4_H_6_NO_3_)_2_(H_2_O)_2_], is a polymorph of the previously reported complex [Dudarenko *et al.* (2010 ▶). *Acta Cryst.* E**66**, m277–m278]. The Ni^II^ atom, lying on an inversion center, is six-coordinated by two carboxyl­ate O atoms and two oxime N atoms from two *trans*-disposed chelating 3-hydroxy­imino­butanoate ligands and two axial water mol­ecules in a distorted octa­hedral geometry. The hydr­oxy group forms an intra­molecular hydrogen bond with the coordinated carboxyl­ate O atom. The complex mol­ecules are linked in stacks along [010] by a hydrogen bond between the water O atom and the carboxyl­ate O atom of a neighboring mol­ecule. The stacks are further linked by O—H⋯O hydrogen bonds into a layer parallel to (001).

## Related literature

For the monoclinic polymorph of the title compound, see: Dudarenko *et al.* (2010 ▶). For the coordination chemistry of hydroxy­imino­carboxylic acids and their derivatives, see: Duda *et al.* (1997 ▶); Mokhir *et al.* (2002 ▶); Moroz *et al.* (2008 ▶); Onindo *et al.* (1995 ▶). For 2-hydroxy­imino­carboxylic acids as efficient metal chelators, see: Gumienna-Kontecka *et al.* (2000 ▶); Sliva *et al.* (1997*a* ▶,*b* ▶). For the use of 2-hydroxy­imino­carboxylic acid derivatives as efficient ligands for stabilization of high oxidation states of transitional metals, see: Fritsky *et al.* (2006 ▶); Kanderal *et al.* (2005 ▶). For structures with monodentately coordinated carboxyl­ate groups, see: Wörl *et al.* (2005*a* ▶,*b* ▶). For the ligand synthesis, see: Khromov (1950 ▶).
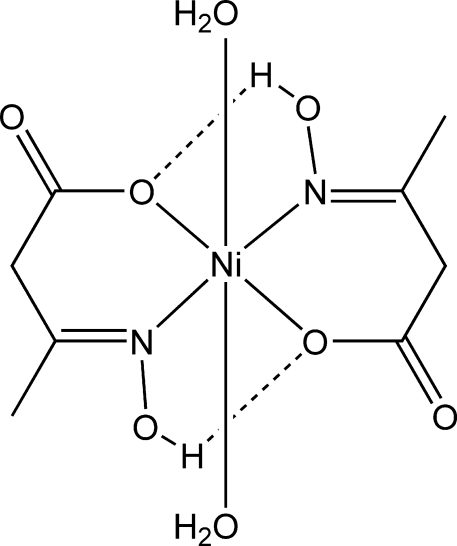


## Experimental

### 

#### Crystal data

[Ni(C_4_H_6_NO_3_)_2_(H_2_O)_2_]
*M* *_r_* = 326.92
Triclinic, 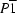

*a* = 5.5621 (14) Å
*b* = 7.340 (2) Å
*c* = 8.2979 (15) Å
α = 90.71 (2)°
β = 92.290 (18)°
γ = 112.18 (2)°
*V* = 313.31 (14) Å^3^
*Z* = 1
Mo *K*α radiation
μ = 1.59 mm^−1^
*T* = 120 K
0.22 × 0.14 × 0.10 mm

#### Data collection

Nonius KappaCCD diffractometer
Absorption correction: multi-scan (*SADABS*; Sheldrick, 1996 ▶) *T*_min_ = 0.764, *T*_max_ = 0.856
2755 measured reflections
1223 independent reflections
1054 reflections with *I* > 2σ(*I*)
*R*_int_ = 0.063

#### Refinement

*R*[*F* ^2^ > 2σ(*F* ^2^)] = 0.036
*wR*(*F* ^2^) = 0.083
*S* = 0.98
1223 reflections
101 parameters
H atoms treated by a mixture of independent and constrained refinement
Δρ_max_ = 0.37 e Å^−3^
Δρ_min_ = −0.35 e Å^−3^

### 

Data collection: *COLLECT* (Nonius, 1998 ▶); cell refinement: *DENZO*/*SCALEPACK* (Otwinowski & Minor, 1997 ▶); data reduction: *DENZO*/*SCALEPACK*; program(s) used to solve structure: *SIR2004* (Burla *et al.*, 2005 ▶); program(s) used to refine structure: *SHELXL97* (Sheldrick, 2008 ▶); molecular graphics: *ORTEP-3* (Farrugia, 1997 ▶); software used to prepare material for publication: *SHELXL97*.

## Supplementary Material

Crystal structure: contains datablocks global, I. DOI: 10.1107/S1600536810006306/hy2284sup1.cif

Structure factors: contains datablocks I. DOI: 10.1107/S1600536810006306/hy2284Isup2.hkl

Additional supplementary materials:  crystallographic information; 3D view; checkCIF report

## Figures and Tables

**Table 1:** Hydrogen-bond geometry (Å, °)

*D*—H⋯*A*	*D*—H	H⋯*A*	*D*⋯*A*	*D*—H⋯*A*
O3—H1*O*⋯O1^i^	0.75 (4)	2.14 (4)	2.766 (3)	142 (4)
O4—H4*O*1⋯O2^ii^	0.79 (5)	2.07 (5)	2.851 (3)	169 (5)
O4—H4*O*1⋯O1^ii^	0.79 (5)	2.50 (5)	3.068 (3)	130 (4)
O4—H4*O*2⋯O2^iii^	0.77 (5)	2.00 (5)	2.754 (3)	166 (4)

Symmetry codes: (i) 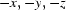; (ii) 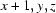; (iii) 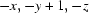.

**Fig. 1.**
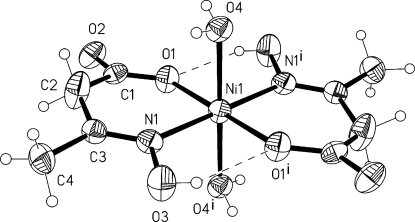
Molecular structure of the title compound, with displacement ellipsoids shown at the 50% probability level. Hydrogen bonds are indicated by dashed lines. [Symmetry code: (i) -x, -y, -z.]

**Fig. 2.**
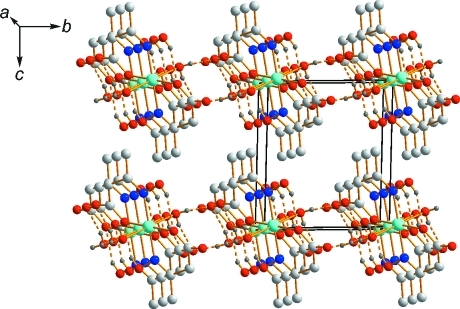
A packing diagram of the title compound. Hydrogen bonds are indicated by dashed lines. H atoms not involved in hydrogen bonding have been omitted for clarity.

## supplementary crystallographic information

### Comment

2-Hydroxyiminocarboxylates of various metal ions are an interesting group of chelate complexes intensively studied during the past 15 years (Duda *et al.*, 1997; Onindo *et al.*, 1995). It was shown that 2-hydroxyiminopropanoic acid and other 2-hydroxyiminocarboxylic acids act as efficient chelators with respect to copper(II), nickel(II) and aluminium(III) (Gumienna-Kontecka *et al.*, 2000; Onindo *et al.*, 1995; Sliva *et al.*, 1997a,b). The amide derivatives of 2-hydroxyiminopropanoic acid have been successfully used for the synthesis of metal complexes with efficient stabilization of trivalent oxidation state of Ni and Cu (Fritsky *et al.*, 2006; Kanderal *et al.*, 2005). Recently we reported the first crystal structure of a metal compound of the nearest homologue of 2-hydroxyiminopropanoic acid, 3-hydroxyiminobutanoic acid, a mononuclear complex with Ni (Dudarenko *et al.*, 2010). In the course of our synthetic study we found that a slight change of experimental conditions, namely use of nickel(II) sulfate instead of nickel(II) nitrate and conduction of synthesis at room temperature resulted in crystallization of a polymorph modification of the title compound.

A distorted octahedral coordination geometry is found in the title complex with the Ni^II^ atom lying on an inversion center (Fig. 1). Two O atoms and two N atoms from two chelating ligands define the equatorial plane, each ligand defining a six-membered ring with a nearly planar conformation, and the two *trans*-coordinated water molecules complete the octahedral coordination geometry. The Ni—O bond lengths [1.992 (2) Å] in the equatorial plane are somewhat shorter than the Ni—N bond lengths [2.025 (2) Å]. The O atoms of the protonated oxime group form intramolecular hydrogen bonds with the coordinated carboxylate O atoms, forming five-membered rings and thus fusing two six-membered chelate rings in a pseudomacrocyclic structure. The difference in C—O bond lengths for the coordinated and noncoordinated O atoms [1.271 (2) and 1.250 (2) Å] is typical for monodentately coordinated carboxylate groups (Wörl *et al.*, 2005a,b). The C═N, C═O and N—O bond lengths are typical for 2-hydroxyiminopropanoic acid and its derivatives (Mokhir *et al.*, 2002; Moroz *et al.*, 2008; Onindo *et al.*, 1995; Sliva *et al.*, 1997a,b). In general, the geometrical parameters of the molecule are very close to those observed in the structure of the monoclinic modification of the title complex (Dudarenko *et al.*, 2010).

The octahedral complex molecules are organized in the piles disposed along the *b* axis due to a hydrogen bond formed between the axial water molecule and noncoordinated carboxylate O atom of a neighboring molecule (Table 1). The Ni···Ni separation in the piles is equal to the unit cell parameter *b*. The piles are united in walls with the help of a hydrogen bond of different type (a bifurcate hydrogen bond formed between the water molecule and both coordinated and noncoordinated carboxylate O atoms belonging to a translational molecule). The walls disposed parallel to (0 0 1) are united in a three-dimensional structure only with the help of van der Waals contacts (Fig. 2).

### Experimental

The title compound was synthesized by adding the solution of nickel(II) sulfate hexahydrate (0.1 mmol, 0.026 g) in water (5 ml) to a solution of 3-hydroxyiminobutanoic acid (0.2 mmol, 0.023 g) in water (5 ml). The resultant mixture was filtered and the dark pink filtrate was left to stand at room temperature. Slow evaporation of the solvent yielded lilac crystals of the title compound (yield 73%). 3-Hydroxyiminobutanoic acid was prepared according to the reported procedure (Khromov, 1950).

### Refinement

O-bound H atoms were located from a difference Fourier map and refined isotropically. H atoms of methyl and methylene groups were positioned geometrically and were constrained to ride on their parent atoms, with C—H = 0.97 (CH_2_) and 0.96 (CH_3_) Å and with *U*_iso_(H) = 1.2(1.5 for methyl)*U*_eq_(C).

### Crystal data

**Table:**

[Ni(C_4_H_6_NO_3_)_2_(H_2_O)_2_]	*Z* = 1
*M**_r_* = 326.92	*F*(000) = 170
Triclinic, *P*1	*D*_x_ = 1.733 Mg m^−^^3^
Hall symbol: -P 1	Mo *K*α radiation, λ = 0.71073 Å
*a* = 5.5621 (14) Å	Cell parameters from 1225 reflections
*b* = 7.340 (2) Å	θ = 3.9–36.0°
*c* = 8.2979 (15) Å	µ = 1.59 mm^−^^1^
α = 90.71 (2)°	*T* = 120 K
β = 92.290 (18)°	Block, dark pink
γ = 112.18 (2)°	0.22 × 0.14 × 0.10 mm
*V* = 313.31 (14) Å^3^	

### Data collection

**Table:**

Nonius KappaCCD diffractometer	1223 independent reflections
Radiation source: fine-focus sealed tube	1054 reflections with *I* > 2σ(*I*)
horizontally mounted graphite crystal	*R*_int_ = 0.063
Detector resolution: 9 pixels mm^-1^	θ_max_ = 26.0°, θ_min_ = 3.8°
φ and ω scans with κ offset	*h* = −6→6
Absorption correction: multi-scan (*SADABS*; Sheldrick, 1996)	*k* = −9→9
*T*_min_ = 0.764, *T*_max_ = 0.856	*l* = −10→10
2755 measured reflections	

### Refinement

**Table:**

Refinement on *F*^2^	Primary atom site location: structure-invariant direct methods
Least-squares matrix: full	Secondary atom site location: difference Fourier map
*R*[*F*^2^ > 2σ(*F*^2^)] = 0.036	Hydrogen site location: inferred from neighbouring sites
*wR*(*F*^2^) = 0.083	H atoms treated by a mixture of independent and constrained refinement
*S* = 0.98	*w* = 1/[σ^2^(*F*_o_^2^) + (0.0431*P*)^2^] where *P* = (*F*_o_^2^ + 2*F*_c_^2^)/3
1223 reflections	(Δ/σ)_max_ < 0.001
101 parameters	Δρ_max_ = 0.37 e Å^−^^3^
0 restraints	Δρ_min_ = −0.35 e Å^−^^3^

### Fractional atomic coordinates and isotropic or equivalent isotropic displacement parameters (Å^2^)

**Table:**

	*x*	*y*	*z*	*U*_iso_*/*U*_eq_	
Ni1	0.0000	0.0000	0.0000	0.0293 (2)	
O1	−0.1803 (4)	0.1846 (3)	0.0358 (2)	0.0363 (5)	
O2	−0.2828 (4)	0.4116 (3)	0.1563 (3)	0.0418 (5)	
O3	0.3067 (5)	−0.0513 (4)	0.2814 (3)	0.0521 (7)	
O4	0.3222 (4)	0.2337 (3)	−0.0904 (3)	0.0349 (5)	
N1	0.1703 (4)	0.0643 (3)	0.2262 (3)	0.0309 (5)	
C1	−0.1519 (5)	0.3071 (4)	0.1501 (3)	0.0298 (6)	
C2	0.0492 (7)	0.3370 (6)	0.2868 (4)	0.0537 (9)	
H2A	0.1902	0.4608	0.2678	0.064*	
H2B	−0.0298	0.3578	0.3840	0.064*	
C3	0.1734 (5)	0.1944 (4)	0.3286 (3)	0.0304 (6)	
C4	0.3120 (7)	0.2237 (5)	0.4912 (3)	0.0452 (8)	
H4A	0.2952	0.0982	0.5328	0.068*	
H4B	0.2369	0.2883	0.5635	0.068*	
H4C	0.4926	0.3033	0.4816	0.068*	
H1O	0.315 (8)	−0.112 (6)	0.210 (5)	0.065 (14)*	
H4O1	0.428 (9)	0.268 (7)	−0.018 (6)	0.081 (16)*	
H4O2	0.284 (8)	0.322 (7)	−0.111 (5)	0.063 (13)*	

### Atomic displacement parameters (Å^2^)

**Table:**

	*U*^11^	*U*^22^	*U*^33^	*U*^12^	*U*^13^	*U*^23^
Ni1	0.0312 (3)	0.0324 (3)	0.0284 (3)	0.0174 (2)	−0.00632 (19)	−0.00037 (19)
O1	0.0400 (12)	0.0406 (12)	0.0364 (10)	0.0259 (10)	−0.0105 (8)	−0.0039 (9)
O2	0.0405 (12)	0.0347 (11)	0.0583 (12)	0.0242 (10)	−0.0057 (10)	−0.0012 (10)
O3	0.0778 (18)	0.0612 (17)	0.0394 (12)	0.0541 (15)	−0.0208 (11)	−0.0069 (11)
O4	0.0372 (13)	0.0324 (12)	0.0378 (11)	0.0169 (10)	−0.0043 (9)	0.0020 (9)
N1	0.0321 (13)	0.0348 (13)	0.0310 (11)	0.0189 (11)	−0.0046 (9)	0.0061 (10)
C1	0.0267 (14)	0.0253 (14)	0.0384 (14)	0.0112 (12)	−0.0005 (11)	0.0044 (11)
C2	0.060 (2)	0.053 (2)	0.058 (2)	0.0357 (18)	−0.0244 (16)	−0.0216 (16)
C3	0.0286 (14)	0.0334 (15)	0.0288 (13)	0.0116 (12)	−0.0020 (11)	0.0017 (11)
C4	0.055 (2)	0.0475 (19)	0.0314 (15)	0.0186 (16)	−0.0112 (13)	−0.0036 (13)

### Geometric parameters (Å, °)

**Table:**

Ni1—O1	1.992 (2)	N1—C3	1.265 (4)
Ni1—N1	2.035 (2)	C1—C2	1.514 (4)
Ni1—O4	2.130 (2)	C2—C3	1.493 (4)
O1—C1	1.262 (4)	C2—H2A	0.9700
O2—C1	1.243 (4)	C2—H2B	0.9700
O3—N1	1.405 (3)	C3—C4	1.499 (4)
O3—H1O	0.75 (4)	C4—H4A	0.9600
O4—H4O1	0.79 (5)	C4—H4B	0.9600
O4—H4O2	0.77 (5)	C4—H4C	0.9600
			
O1^i^—Ni1—O1	180.00 (13)	C3—N1—Ni1	130.1 (2)
O1^i^—Ni1—N1	89.62 (9)	O3—N1—Ni1	116.79 (18)
O1—Ni1—N1	90.38 (9)	O2—C1—O1	122.5 (3)
O1^i^—Ni1—N1^i^	90.38 (9)	O2—C1—C2	116.2 (3)
O1—Ni1—N1^i^	89.62 (9)	O1—C1—C2	121.3 (3)
N1—Ni1—N1^i^	180.00 (15)	C3—C2—C1	124.7 (3)
O1^i^—Ni1—O4^i^	90.13 (9)	C3—C2—H2A	106.1
O1—Ni1—O4^i^	89.87 (9)	C1—C2—H2A	106.1
N1—Ni1—O4^i^	90.34 (9)	C3—C2—H2B	106.1
N1^i^—Ni1—O4^i^	89.66 (9)	C1—C2—H2B	106.1
O1^i^—Ni1—O4	89.87 (9)	H2A—C2—H2B	106.3
O1—Ni1—O4	90.13 (9)	N1—C3—C2	120.3 (2)
N1—Ni1—O4	89.66 (9)	N1—C3—C4	123.3 (3)
N1^i^—Ni1—O4	90.34 (9)	C2—C3—C4	116.3 (3)
O4^i^—Ni1—O4	180.00 (15)	C3—C4—H4A	109.5
C1—O1—Ni1	130.05 (18)	C3—C4—H4B	109.5
N1—O3—H1O	106 (3)	H4A—C4—H4B	109.5
Ni1—O4—H4O1	106 (3)	C3—C4—H4C	109.5
Ni1—O4—H4O2	111 (3)	H4A—C4—H4C	109.5
H4O1—O4—H4O2	107 (4)	H4B—C4—H4C	109.5
C3—N1—O3	113.1 (2)		
			
Ni1—O1—C1—O2	−179.15 (18)	Ni1—N1—C3—C2	−3.3 (4)
Ni1—O1—C1—C2	1.9 (4)	O3—N1—C3—C4	0.8 (4)
O2—C1—C2—C3	162.2 (3)	Ni1—N1—C3—C4	−179.4 (2)
O1—C1—C2—C3	−18.8 (5)	C1—C2—C3—N1	19.2 (5)
O3—N1—C3—C2	176.9 (3)	C1—C2—C3—C4	−164.4 (3)

Symmetry codes: (i) −*x*, −*y*, −*z*.

### Hydrogen-bond geometry (Å, °)

**Table:**

*D*—H···*A*	*D*—H	H···*A*	*D*···*A*	*D*—H···*A*
O3—H1O···O1^i^	0.75 (4)	2.14 (4)	2.766 (3)	142 (4)
O4—H4O1···O2^ii^	0.79 (5)	2.07 (5)	2.851 (3)	169 (5)
O4—H4O1···O1^ii^	0.79 (5)	2.50 (5)	3.068 (3)	130 (4)
O4—H4O2···O2^iii^	0.77 (5)	2.00 (5)	2.754 (3)	166 (4)

Symmetry codes: (i) −*x*, −*y*, −*z*; (ii) *x*+1, *y*, *z*; (iii) −*x*, −*y*+1, −*z*.

## References

[bb1] Burla, M. C., Caliandro, R., Camalli, M., Carrozzini, B., Cascarano, G. L., De Caro, L., Giacovazzo, C., Polidori, G. & Spagna, R. (2005). *J. Appl. Cryst.* **38**, 381–388.

[bb2] Duda, A. M., Karaczyn, A., Kozłowski, H., Fritsky, I. O., Głowiak, T., Prisyazhnaya, E. V., Sliva, T. Yu. & Świątek-Kozłowska, J. (1997). *J. Chem. Soc. Dalton Trans.* pp. 3853–3859.

[bb3] Dudarenko, N. M., Kalibabchuk, V. A., Malysheva, M. L., Iskenderov, T. S. & Gumienna-Kontecka, E. (2010). *Acta Cryst.* E**66**, m277–m278. 10.1107/S1600536810004605 PMC2983569 21580224

[bb4] Farrugia, L. J. (1997). *J. Appl. Cryst.* **30**, 565.

[bb5] Fritsky, I. O., Kozłowski, H., Kanderal, O. M., Haukka, M., Świątek-Kozłowska, J., Gumienna-Kontecka, E. & Meyer, F. (2006). *Chem. Commun* pp. 4125–4127. 10.1039/b608236j 17024270

[bb6] Gumienna-Kontecka, E., Berthon, G., Fritsky, I. O., Wieczorek, R., Latajka, Z. & Kozłowski, H. (2000). *J. Chem. Soc. Dalton Trans* pp. 4201–4208.

[bb7] Kanderal, O. M., Kozłowski, H., Dobosz, A., Świątek-Kozłowska, J., Meyer, F. & Fritsky, I. O. (2005). *Dalton Trans* pp. 1428–1437. 10.1039/b418598f 15824781

[bb8] Khromov, N. V. (1950). *Zh. Obshch. Khim.* **20**, 1858–1867.

[bb9] Mokhir, A. A., Gumienna-Kontecka, E., Świątek-Kozłowska, J., Petkova, E. G., Fritsky, I. O., Jerzykiewicz, L., Kapshuk, A. A. & Sliva, T. Yu. (2002). *Inorg. Chim. Acta*, **329**, 113–121.

[bb10] Moroz, Yu. S., Kulon, K., Haukka, M., Gumienna-Kontecka, E., Kozłowski, H., Meyer, F. & Fritsky, I. O. (2008). *Inorg. Chem.* **47**, 5656–5665. 10.1021/ic702375h 18500796

[bb11] Nonius (1998). *COLLECT* Nonius BV, Delft, The Netherlands.

[bb12] Onindo, C. O., Sliva, T. Yu., Kowalik-Jankowska, T., Fritsky, I. O., Buglyo, P., Pettit, L. D., Kozłowski, H. & Kiss, T. (1995). *J. Chem. Soc. Dalton Trans.* pp. 3911–3915.

[bb13] Otwinowski, Z. & Minor, W. (1997). *Methods in Enzymology*, Vol. 276, *Macromolecular Crystallography*, Part A, edited by C. W. Carter Jr & R. M. Sweet, pp. 307–326. New York: Academic Press.

[bb14] Sheldrick, G. M. (1996). *SADABS* University of Göttingen, Germany.

[bb15] Sheldrick, G. M. (2008). *Acta Cryst.* A**64**, 112–122. 10.1107/S0108767307043930 18156677

[bb16] Sliva, T. Yu., Duda, A. M., Głowiak, T., Fritsky, I. O., Amirkhanov, V. M., Mokhir, A. A. & Kozłowski, H. (1997*a*). *J. Chem. Soc. Dalton Trans* pp. 273–276.

[bb17] Sliva, T. Yu., Kowalik-Jankowska, T., Amirkhanov, V. M., Głowiak, T., Onindo, C. O., Fritsky, I. O. & Kozłowski, H. (1997*b*). *J. Inorg. Biochem.* **65**, 287–294.

[bb18] Wörl, S., Fritsky, I. O., Hellwinkel, D., Pritzkow, H. & Krämer, R. (2005*a*). *Eur. J. Inorg. Chem* pp. 759–765.

[bb19] Wörl, S., Pritzkow, H., Fritsky, I. O. & Krämer, R. (2005*b*). *Dalton Trans* pp. 27–29. 10.1039/b417053a 15605143

